# The Novel Key Genes of Non-obstructive Azoospermia Affect Spermatogenesis: Transcriptomic Analysis Based on RNA-Seq and scRNA-Seq Data

**DOI:** 10.3389/fgene.2021.608629

**Published:** 2021-02-26

**Authors:** Haihong He, Fan Yu, Wang Shen, Keyan Chen, Lijun Zhang, Shuang Lou, Qiaomin Zhang, Siping Chen, Xinhua Yuan, Xingwang Jia, Yiwen Zhou

**Affiliations:** ^1^Department of Emergency Laboratory, Clinical Laboratory Medical Center, Shenzhen Hospital, Southern Medical University, Shenzhen, China; ^2^Department of Clinical Laboratory, Affiliated Jiangmen TCM Hospital of Ji’nan University, Jiangmen, China

**Keywords:** non-obstructive azoospermia, spermatogenesis, scRNA-seq, differentially express genes, TSSK6

## Abstract

Non-obstructive azoospermia (NOA) is one of the most important causes of male infertility. It is mainly characterized by the absence of sperm in semen repeatedly or the number of sperm is small and not fully developed. At present, its pathogenesis remains largely unknown. The goal of this study is to identify hub genes that might affect biomarkers related to spermatogenesis. Using the clinically significant transcriptome and single-cell sequencing data sets on the Gene Expression Omnibus (GEO) database, we identified candidate hub genes related to spermatogenesis. Based on them, we performed Gene Ontology (GO) functional enrichment analysis, Kyoto Encyclopedia of Genes and Genomes (KEGG) enrichment pathway analyses, protein-protein interaction (PPI) network analysis, principal component analysis (PCA), cell cluster analysis, and pseudo-chronological analysis. We identified a total of 430 differentially expressed genes, of which three have not been reported related to spermatogenesis (C22orf23, TSACC, and TTC25), and the expression of these three hub genes was different in each type of sperm cells. The results of the pseudo-chronological analysis of the three hub genes indicated that TTC25 was in a low expression state during the whole process of sperm development, while the expression of C22orf23 had two fluctuations in the differentiating spermatogonia and late primary spermatocyte stages, and TSACC showed an upward trend from the spermatogonial stem cell stage to the spermatogenesis stage. Our research found that the three hub genes were different in the trajectory of sperm development, indicating that they might play important roles in different sperm cells. This result is of great significance for revealing the pathogenic mechanism of NOA and further research.

## Introduction

About 10–15% of people of childbearing age are infertile in the world, of which male infertility accounts for about 50% (Gifford, 2015). Male infertility is closely related to sexual dysfunction, varicocele, reproductive system infection, endocrine, obstructive azoospermia (OA), non-obstructive azoospermia (NOA), etc. (Arafat et al., 2017). The incidence of NOA in men is about 1%, accounting for 10–15% of infertile men, and it is one of the most important causes of male infertility (Wosnitzer et al., 2014).

Non-obstructive azoospermia is a type of male infertility caused by spermatogenic dysfunction of testicular tissue. Patients with NOA cannot produce sperm or can only produce a very small amount of sperm. In patients with NOA, the structure of the seminiferous tubules in the testis is disordered, while the maturation of spermatogenic cells is blocked, and the meiosis of spermatogenic cells is arrested (Kohn and Pastuszak, 2018). In recent years, some studies (Vij et al., 2018) have shown that there are focal and heterogeneous tissues in the spermatogenesis disorder. Even if sperm are not found in most of the seminiferous tubules in the testicular tissue, it cannot be completely denied that there may be a very small amount of sperm in some seminiferous tubules. Studies have confirmed that some sperm found from the testis or epididymis in patients whose spermatogenic cells stop during meiosis can also be conceived by intracytoplasmic sperm injection (ICSI) diagnosis and treatment (Silber et al., 1996). There are also studies reporting that the use of round or long sperm can also enable patients to gain fertility (Sofikitis et al., 1998; Ghazzawi et al., 1999). Reproductive technology has greatly reduced requirements for sperm quantity and sperm maturity compared with natural conception conditions, so that it can become a reality for NOA patients to obtain genetic offspring (Corona et al., 2019; Song et al., 2020). Because reproductive technology bypasses the natural selection mechanism in the process of sperm formation, the risk of genetic defects being passed to the next generation is also significantly increased. Therefore, it is particularly important to grasp the key factors that affect the process of sperm development.

At present, the research on NOA mainly focuses on including chromosomal abnormalities, Y chromosome microdeletion, and epigenetics. However, the spermatogenesis barriers and pathogenesis related to gene expression levels are still to be explored (Fang et al., 2020). In this study, we mainly used the gene expression profile microarray transcriptome data and single cell sequencing data in the NCBI Gene Expression Comprehensive Database (NCBI-GEO) database. By analyzing transcriptome data, we identified differentially expressed genes (DEGs) between patients with and without spermatogenesis disorders. Subsequently, we performed Gene Ontology (GO) function enrichment analysis, Kyoto Encyclopedia of Genes and Genomes (KEGG) enrichment pathway analysis, protein-protein interaction (PPI) network analysis, and identification of hub genes. By analyzing the sperm single-cell sequencing data, we annotated the cell clusters, revealing the expression of the hub gene in the cell clusters, and finally performed a pseudo-chronological analysis of the hub gene to reveal the role of the hub gene in sperm development.

## Materials And Methods

### Data Source

Gene Expression Omnibus (GEO) is a public genome database that provides gene expression data, microarray, and single cell sequencing data.^1^ The transcriptome data of this study were from two datasets: GSE108886 and GSE145467. People without spermatogenesis disorders were included in the control group, including normal people and patients with OA, and patients with NOA were included in the disease group. The population of the GSE108886 data set consists of one normal person, three OA patients and eight NOA patients. The GSE145467 data set consists of 10 OA patients and 10 NOA patients. The single-cell sequencing data were from the GSE109037 data set, which contained 11 samples from four sub-data sets (spermatogenesis, spermatocytes, spermatogonia, and spermatids) during sperm development.

### Identification of Differentially Expressed Genes

The GEO2R online analysis software on GEO database was used to identify DEGs,^2^ and the screening threshold was set to |log2 FC| > 2 and *p* < 0.01.

### Functional Enrichment Analysis of the DEGs

The DAVID online analysis tool was used for GO and KEGG enrichment analyses of DEGs.^3^ We then use the imageGP tool for visual analysis.^4^ *p* < 0.05 was considered statistically significant.

### Construction of PPI Network and Identification of Hub Genes

We use the String database to build the PPI network of DEGs,^5^ and set the interaction with combined score to be greater than or equal to 0.4. Subsequently, the data after String analysis were imported into Cytoscape software (version 3.6.1) to construct the PPI network again. The five calculation methods (Degree, DMNC, EPC, MCC, and MNC) in the CytoHubba plug-in were used to identify hub genes in the PPI network. A total of 50 hub genes (TOP10 in each algorithm) were selected to be involved in the Veen Diagram, and the core hub genes were then identified for subsequent analysis.

### Cell Clustering and Annotation of Single Cell Sequencing Data

We used the Seurat package (version 3.1.5.9915) of the R software (version 4.02) to analyze the four sub-sets of GSE109037. After a series of data filtering, quality control, and normalization, we performed principal component analysis (PCA) and non-linear dimensionality reduction UMAP method for cell clustering. Through the FindAllMarkers function, we obtained the marker genes of the cell clustering, and then we queried the Human Cell Landscape (HPL) database for cell annotation.^6^ The DotPlot and VlnPlot functions were used to draw bubble charts and violin charts to visualize the expression of hub genes in different cell clusters.

### Constructing Trajectories of Hub Genes in Single Cell

The monocle3 package (version 0.2.3.0) was used to perform pseudotime time analysis to construct the developmental trajectory of sperm cells and hub genes. The main functions used were learn_graph, label_leaves, label_branch_points, plot_cells, order_cells, and root_pr_node.

## Results

### Identification of DEGs

**Figure 1:**
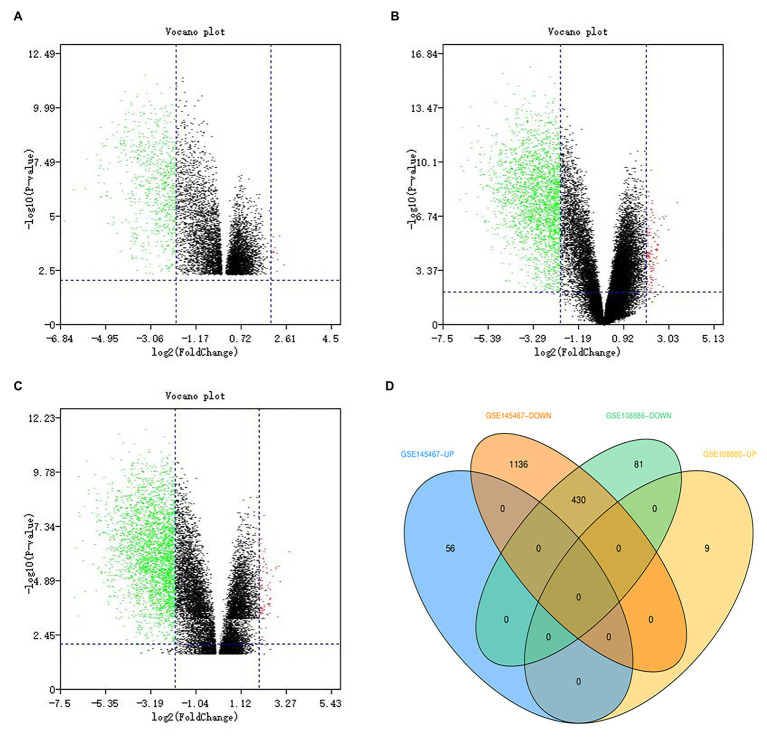
Identification of differentially expressed genes (DEGs). Green dots represent downregulated genes, and red dots represent upregulated genes. **(A)** The volcano map showing DEGs in the GSE108886 data set. **(B)** The volcano map showing DEGs in the GSE145467 data set. **(C)** The volcano map showing DEGs after data set integration. **(D)** The Venn diagram showing that there are 430 DEGs overlapping these two data sets.

In the GSE108886 data set, a total of 520 DEGs were obtained, including 511 downregulated genes and nine upregulated genes (Figure 1A), while in the GSE145467 data set, a total of 1,622 DEGs were obtained, including 1,566 downregulated genes and 56 upregulated genes (Figure 1B). There were 430 DEGs overlapping the two data sets, and all of them were downregulated genes (Figures 1C,D).

### GO Functional Enrichment Analysis and KEGG Pathway Analysis

**Figure 2:**
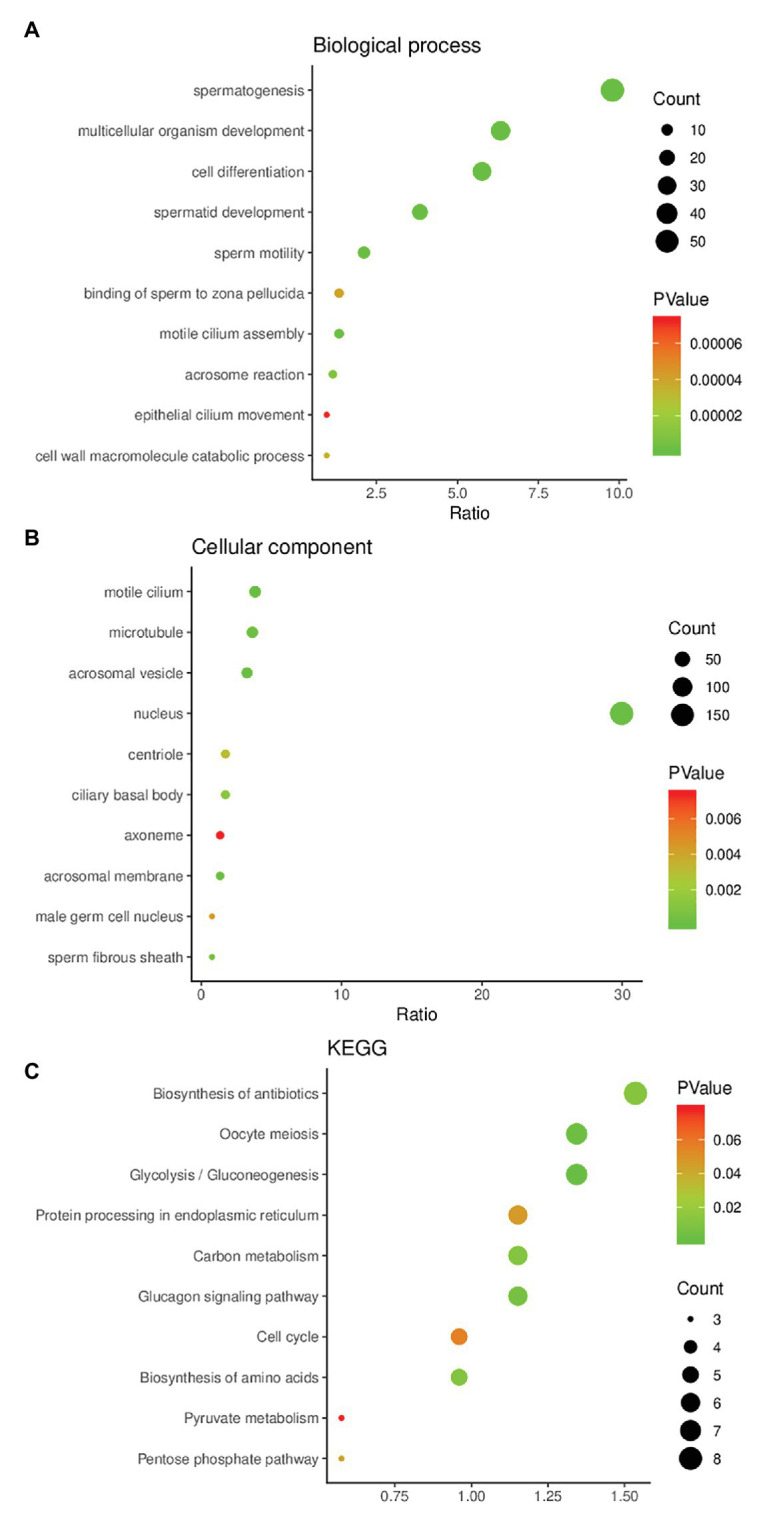
Gene Ontology (GO) enrichment analysis and Kyoto Encyclopedia of Genes and Genomes (KEGG) pathway enrichment analysis of DEGs. **(A)** The result of GO enrichment analysis (biological process). **(B)** The result of GO enrichment analysis (cellular component). **(C)** The result of KEGG pathway enrichment analysis.

The results of GO functional enrichment analysis showed that the DEGs were mainly enriched in biological processes (BP) and cell composition (CC). In the BP category, they were mainly enriched in spermatogenesis, multicellular organism development, cell differentiation, spermatid development, and sperm motility (Figure 2A). In the CC category, they were mainly enriched in modile cilium, microtubule, acrosomal vesicle, and nucleus (Figure 2B). In KEGG pathway analysis, we found that these candidate genes might affect spermatogenesis signaling pathways including glycolysis/gluconeogenesis, protein processing in endoplasmic reticulum, carbon metabolism, and cell cycle (Figure 2C).

### Construction of PPI Network of DEGs and Identification of Hub Genes

**Figure 3:**
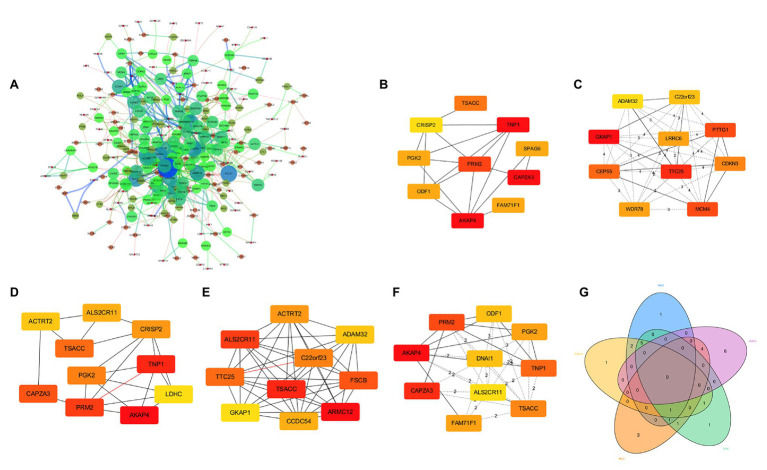
Protein-protein interaction (PPI) network of DEGs and identification of hub genes. **(A)** PPI network composed of 304 nodes and 933 edges. **(B)** Hub genes obtained by Degree algorithm. **(C)** Hub genes obtained by Density of Maximum Neighborhood Component algorithm. **(D)** Hub genes obtained by Edge Percolated component algorithm. **(E)** Hub genes obtained by Maximal Clique Centrality algorithm. **(F)** Hub genes obtained by Maximum Neighborhood Component algorithm. **(G)** Venn diagram showing 15 overlapped hub genes.

We mapped these DEGs to the STRING database to construct a PPI network, and then re-imported these interactive network data into Cytoscape software (version 3.6.0), and finally 304 nodes and 933 edges constituted the PPI network (Figure 3A). We used five algorithms in cytoHubba to obtain the top 10 hub genes of each algorithm (Figures 3B–F). Then, these hub genes were integrated, and 15 of them overlapped in the five algorithms (ACTRT2, ADAM32, AKAP4, ALS2CR11, C22orf23, CAPZA3, CRISP2, FAM71F1, GKAP1, ODF1, PGK2, PRM2, TNP1, TSACC, and TTC25). The 15 hub genes were considered to be key genes (Figure 3G), indicating that they might play important roles in spermatogenesis. After consulting relevant literature (Choi et al., 2003; Malcher et al., 2013; Liu et al., 2015; Javadian-Elyaderani et al., 2016; Malla and Bhandari, 2017; Park et al., 2018; Lim et al., 2019; Tapia Contreras and Hoyer-Fender, 2019; Wang et al., 2019a; Oud et al., 2020; Zhang et al., 2020), among those 15 genes, we screened out three hub genes (C22orf23, TSACC, and TTC25) that have not been reported to be related to spermatogenesis.

### Expression of the Hub Genes in Sperm Cells

**Figure 4:**
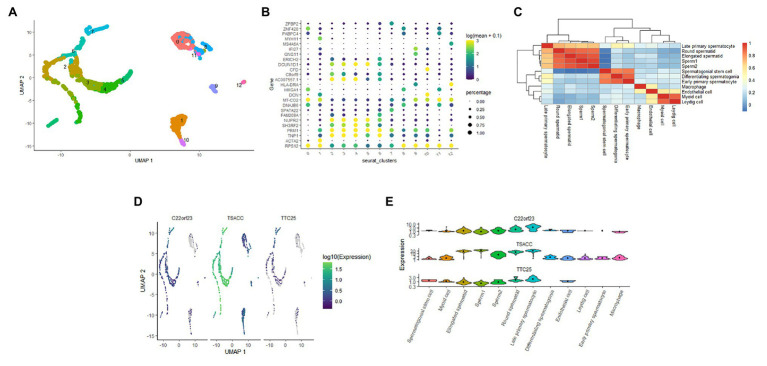
The expression of hub genes in sperm cells. **(A)** Thirteen cell clusters (0–12) of the GSE109037 single-cell sequencing data set. **(B)** The bubble chart showing the marker gene expression of each cell clusters. **(C)** The heat map showing the correlation between each cell clusters. **(D)** Distribution of three hub genes in cell clusters. **(E)** The violin diagram showing the expression of three hub genes in various sperm cells.

To clarify the expression of these three hub genes in different types of sperm cells, we analyzed the single-cell sequencing data covering the whole process of sperm development. Through the Seurat package nonlinear dimensionality reduction method (UMAP), a total of 13 cell clusters were found (Figure 4A). Next, we use the FindAllMarkers function to identify and visualize the marker genes of cell clusters (Figure 4B). By consulting the HPL database through the marker gene, 12 types of sperm cells were identified, and the relationship between them was shown in a heat map (Figure 4C). The analysis results showed that the three hub genes were expressed in late primary spermatocyte, round spermatid, elongated spermatid, sperm1, and sperm2, while testis-specific serine kinase 6 activating co-chaperone (TSACC) had the highest expression level among the three hub genes. In early primary spermatocyte and differentiating spermatogonia cells, TTC25 was almost not expressed, while C22orf23 was slightly expressed, and TSACC expression was higher (Figures 4D,E). This result indicated that there were differences in the expression of hub genes in the development of sperm cells.

### Pseudotime Time Analysis of Hub Genes in Sperm Cells

**Figure 5:**
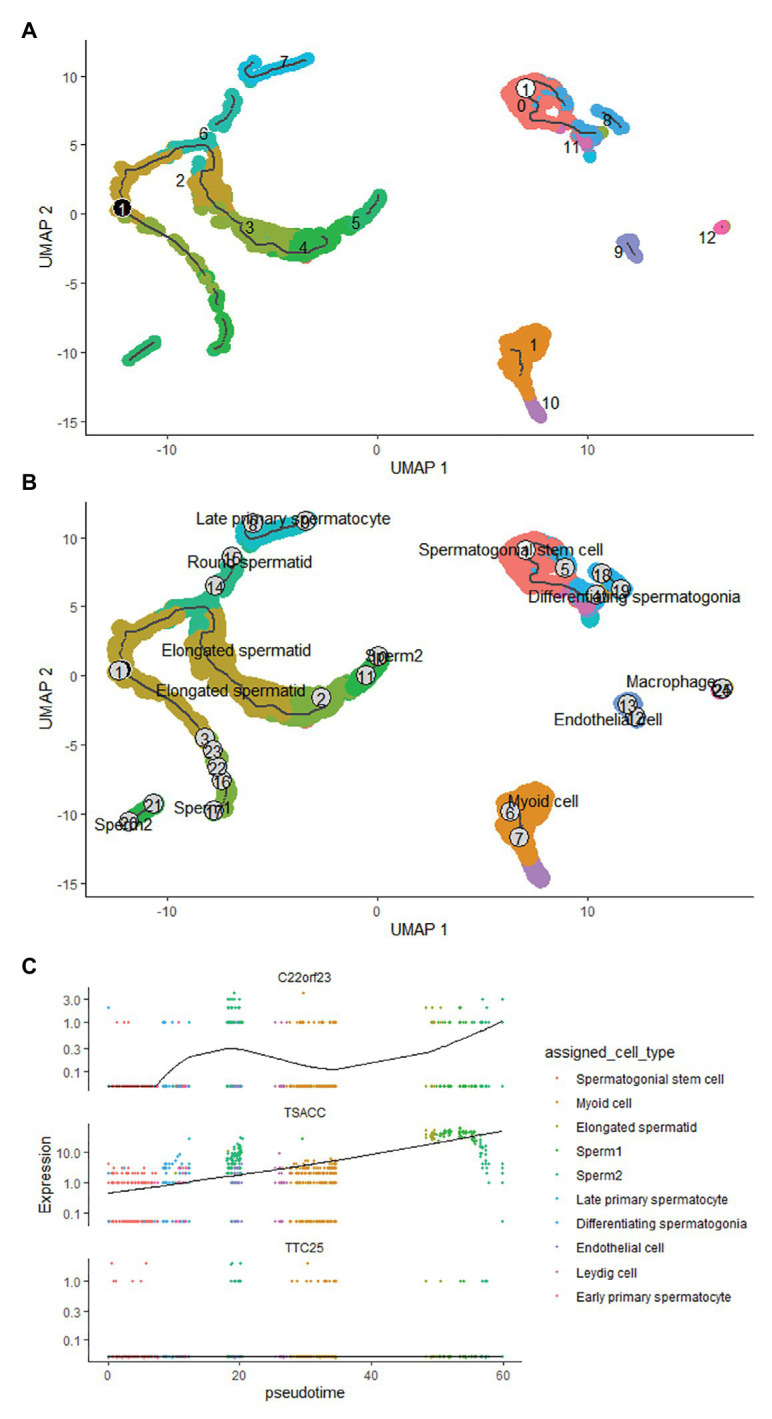
The pseudotime time analysis of sperm development. **(A)** The developmental trajectory of sperm cells. The ① on the white background indicates that the development of sperm starts from cluster 0 cells, and the ① on the black background indicates branches that appear in the sperm development trajectory. **(B)** The sperm cells of each developmental stage are marked, the numbers indicate the sequence of each sperm cell’s development trajectory, and words indicate the result of annotating the clustered cells. **(C)** The results of trajectory analysis of the three hub genes in sperm development, which covers the whole process from spermatogonial stem cell to sperm formation.

The developmental trajectory of sperm cells was constructed using monocle 3 software. The results showed that during sperm development, the initial cell of sperm development was identified as spermatogonial stem cell, and the only branch appeared in the elongated spermatid (Figures 5A,B). We constructed the trajectory of C22orf23, TSACC, and TTC25 in sperm development, and found that TTC25 has been maintained at a low level, while C22orf23 began to rise from the differentiating spermatogonia and the first peak appeared in the late primary spermatocyte, and then gradually decreased, there was an upward trend in the process from elongated spermatid to sperm formation. During the whole process from spermatogonial stem cell to the sperm formation, the expression of TSACC showed an upward trend (Figure 5C).

## Discussion

The process of spermatogenesis is divided into three stages: mitosis stage, meiosis stage, and sperm cell forming stage. Subsequently, mature sperm are freely released into the lumen of seminiferous tubules, and migrate to the epididymal tissue for energy and storage (Neto et al., 2016). In the whole process of spermatogenesis, any abnormality in any link can lead to NOA, which can lead to male infertility (La and Hobbs, 2019). The process of spermatogenesis is a complex process involving a variety of regulatory mechanisms, among which the regulation of gene transcription level has been a hotspot of research in recent years (Singh et al., 2019).

A number of studies have shown (Jan et al., 2017; Law et al., 2019; Trigg et al., 2019) that changes in transcriptome genes can affect the development of different types of sperm cells and ultimately lead to the occurrence of NOA. Our analysis of GSE108886 and GSE145467 found that 430 DEGs were identified in both data sets at the same time and all were downregulated. Subsequent GO analysis showed that these genes were mainly enriched in BP category including spermatogenesis, multicellular organism development, cell differentiation, spermatid development, and sperm motility, as well as in CC category including motile cilium, microtubule, acrosomal vesicle and nucleus. These are also the hotspots of research on male infertility from the transcriptome level in recent years (Moye et al., 2019; Wang et al., 2019b; Yuan et al., 2019; Goto, et al., 2020). KEGG pathway analysis results indicated that the pathways that DEGs might affect sperm development were mainly enriched in glycolysis/gluconeogenesis, protein processing in endoplasmic reticulum, carbon metabolism and cell cycle. Studies have shown that One-carbon metabolism may affect spermatogenesis and lead to male infertility (Singh and Jaiswal, 2013). The protein processing in endoplasmic reticulum signaling pathway affects histone ubiquitination and acetylation during sperm formation, which is crucial for sperm development (Kavarthapu et al., 2020). In proteomic studies, it was found that the glycolysis/gluconeogenesis pathway is different between normal people and asthenospermia patients (Saraswat et al., 2017), but till now, there is no report on the association between glycolysis/gluconeogenesis and NOA patients.

In a total of 50 hub genes obtained by five different algorithms in the cytoHubba plug-in, we obtained 15 genes that overlap in multiple algorithms. After consulting database documents such as NCBI, for the first time, we identified three genes that might be closely related to spermatogenesis: C22orf23, TSACC, and TTC253.

In order to further study the expression of C22orf23, TSACC, and TTC25 in sperm cells, we used the seurat package of the R software to integrate and analyze the corresponding single-cell sequencing data sets. After comparing the data of the HCL database (Han et al., 2020), we identified 12 kinds of sperm cells. Our research results showed that during spermatogenesis, hub genes were expressed in late primary spermatocyte, round spermatid, elongated spermatid, and sperm. The expression of TSACC was of the highest level. In differentiating spermatogonia and early primary spermatocyte cells, TTC25 was almost not expressed, while C22orf23 was lowly expressed, and expression of TSACC is higher than the former two. The above research showed that the expression of the three genes was different in the process of sperm development, and there might be differences in their mechanisms that affect spermatogenesis. The potential mechanisms of spermatogenesis mainly include: (1) the functional structure and cell composition of spermatogenesis, such as blood testis barrier, seminiferous tubules, spermatogenic cells, and testicular somatic cells, etc.; (2) the whole process of spermatogenesis and sperm kinetics of occurrence; (3) endocrine regulation of spermatogenesis; (4) testicular local regulation of spermatogenesis, etc. (Caroppo and Colpi, 2021; Park and Pang, 2021; Rodriguez-Casuriaga and Geisinger, 2021). Differentiating spermatogonia and early primary spermatocyte belong to the representative cells of spermatogenesis during meiosis, so the difference in the expression of these three genes is mainly reflected in the meiosis stage.

Sperm cells have clear developmental trajectories. We used the monocle3 package to successfully construct the developmental trajectory of sperm cells in the GSE109037 dataset. The spematogonial stem cell was recognized as the starting point of spermatogenesis, and a branch was found in the elongated spermatid. The trajectory of C22orf23, TSACC, and TTC25 genes in sperm development was constructed, and it was found that the expression of TTC25 gene was always maintained at a low level. The C22orf23 gene showed two uplifts during the process from differentiating spermatogonia to sperm production. The TSACC gene showed a slow upward trend during the whole process from spermatogonial stem cell to sperm formation. At present, there are few literatures on the correlation between sperm development and C22orf23 gene. However, the TTC25 gene encodes a tetratricopeptide repeat domain-containing protein, which is located in the ciliary axons and plays a role in the docking of the outer actin arm with the cilia. Mutations in this gene can cause primary ciliary dyskinesia (Wallmeier et al., 2016; Emiralioğlu et al., 2020), but whether it is related to sperm development remains to be further studied. TSACC gene is also called testis-specific serine kinase 6-Activating Co-Chaperone Protein (TSSK6-Activating Co-Chaperone), and TSSK6 belongs to a member of the testis-specific serine/threonine kinase family. Studies have confirmed that TSSK family is expressed after meiosis in male germ cells and mature mammalian sperm. When TSSK family expression is restricted after meiosis, it can affect sperm development through phosphorylation signal transduction (Wang et al., 2015, 2016; Salicioni et al., 2020). Animal experiments have confirmed (Spiridonov et al., 2005) that male mice knocked out of the TSSK6 gene can cause spermatogenesis disorders, including decreased sperm count, decreased motility and survival rate, and increased number of abnormal sperm. Su et al. (2010) studied the relationship between TSSK6 gene mutation and human spermatogenesis and found that TSSK6 gene polymorphism is closely related to male infertility. TSACC is a Co-Chaperone that activates TSSK6, and is closely related to the expression of TSSK6. Based on our analysis results, we speculate that TSACC plays an important role in germ cell differentiation and/or sperm function. TSACC is a Co-Chaperone that specifically activates TSSK6 (Spiridonov et al., 2005). Kula and other scholars (Jha et al., 2010) have shown that TSACC is specifically expressed in the testis, and experiments have confirmed that when sperm cells undergo reorganization and chromatin condensation, TSACC and TSSK6 are in sperm co-localization in the cytoplasm of cells, combined with our analysis results, speculate that TSACC plays an important role in germ cell differentiation.

Non-obstructive azoospermia is one of the most common and important causes of male infertility. Our research used existing public databases to integrate gene expression profile microarray and single-cell sequencing data for bioinformatics analysis. In this way, more reliable and accurate results could be obtained. However, to confirm the relationship between these genes and spermatogenesis, molecular biology experiments are needed.

## Conclusion

This study identified 430 DEGs in NOA patients, as well as their GO functional annotations and signaling pathways, and further identified three hub genes that might play important roles in sperm development, which deserved further in-depth study. These new findings may provide important enlightenment for revealing the pathogenesis of NOA. These hub genes may be biomarkers suggesting abnormal spermatogenesis, providing novel options for precise diagnosis and precise treatment of NOA.

## Data Availability Statement

The original contributions presented in the study are included in the article/Supplementary Material, further inquiries can be directed to the corresponding author.

## Author Contributions

HH, YZ, XJ, and FY launched the study. HH and FY performed the data analysis. WS, KC, LZ, QZ, SL, XY, and SC participated in reference collecting. HH, FY, and WS completed the manuscript. All authors contributed to the article and approved the submitted version.

### Conflict of Interest

The authors declare that the research was conducted in the absence of any commercial or financial relationships that could be construed as a potential conflict of interest.

## Footnotes

^1^http://www.ncbi.nlm.nih.gov/geo

^2^http://www.ncbi.nlm.nih.gov/geo/geo2r/

^3^https://david.ncifcrf.gov/

^4^http://www.ehbio.com/ImageGP/index.php/Home

^5^http://string-db.org

^6^http://bis.zju.edu.cn/HCL/
